# Feasibility of cerebello-cortical stimulation for intraoperative neurophysiological monitoring of cerebellar mutism

**DOI:** 10.1007/s00381-021-05126-7

**Published:** 2021-04-09

**Authors:** Davide Giampiccolo, Federica Basaldella, Andrea Badari, Giovanna Maddalena Squintani, Luigi Cattaneo, Francesco Sala

**Affiliations:** 1 Department of Neurosciences, Biomedicine and Movement Sciences, Section of Neurosurgery, University Hospital, Piazzale Stefani 1, 37124 Verona, Italy; 2 Intraoperative Neurophysiology Unit, Division of Neurology, University Hospital, Verona, Italy; 3 Division of Neurology, University Hospital, Verona, Italy; 4 CIMeC-Center for Mind/Brain Sciences, University of Trento, Trento, Italy

**Keywords:** Cerebellar mutism, Intraoperative neurophysiological monitoring, Pediatric brain tumours, Posterior fossa

## Abstract

**Background:**

Cerebellar mutism can occur in a third of children undergoing cerebellar resections. Recent evidence proposes it may arise from uni- or bilateral damage of cerebellar efferents to the cortex along the cerebello-dento-thalamo-cortical pathway. At present, no neurophysiological procedure is available to monitor this pathway intraoperatively. Here, we specifically aimed at filling this gap.

**Methods:**

We assessed 10 patients undergoing posterior fossa surgery using a conditioning-test stimulus paradigm. Electrical conditioning stimuli (cStim) were delivered to the exposed cerebellar cortex at interstimulus intervals (ISIs) of 8–24 ms prior to transcranial electric stimulation of the motor cortex, which served as test stimulus (tStim). The variation of motor-evoked potentials (MEP) to cStim + tStim compared with tStim alone was taken as a measure of cerebello-cortical connectivity.

**Results:**

cStim alone did not produce any MEP. cStim preceding tStim produced a significant inhibition at 8 ms (*p* < 0.0001) compared with other ISIs when applied to the lobules IV-V-VI in the anterior cerebellum and the lobule VIIB in the posterior cerebellum. Mixed effects of decrease and increase in MEP amplitude were observed in these areas for longer ISIs.

**Conclusions:**

The inhibition exerted by cStim at 8 ms on the motor cortex excitability is likely to be the product of activity along the cerebello-dento-thalamo-cortical pathway. We show that monitoring efferent cerebellar pathways to the motor cortex is feasible in intraoperative settings. This study has promising implications for pediatric posterior fossa surgery with the aim to preserve the cerebello-cortical pathways and thus prevent cerebellar mutism.

## Introduction

Brain tumours represent the most common solid tumour in the pediatric population, with the majority of them being located in the posterior fossa [1, 2]. Initially described in 1958 [3], cerebellar mutism (CM, also referred as akinetic mutism or posterior fossa syndrome) includes a variety of signs and symptoms including mutism or speech disturbances, dysphagia, decreased attention and emotional lability. Despite being only anecdotal reported before 1995 [4], recent publication shows that it may occur from 26.6 to 32% of pediatric patients, with up to 39% of patients operated for medulloblastoma [5]. While mutism is always transient [6], short-term recovery is incomplete in 98.8% of patients [7]. Long-term language and cognitive deficits have been documented in a not negligible number of patients [8–10].

The prevalence of non-motor symptoms of CM is consistent with the hypothesis of cerebello-cortical connections responsible for a cerebellar modulation of cognitive functions [11]. While in the past, a role for cerebello-cortical pathways was restricted to motor control, there is now extensive evidence that the cerebellum also exerts non-motor functions [12], with the cerebellar cortex as well as its subcortical nuclei playing a role in cognition [12–15]. The leading hypothesis for the occurrence of CM is mono- or bilateral disconnection of the efferent pathway from the cerebellum to the cortex, namely the cerebello-dento-thalamo-cortical (CDTC) pathway [16]. This has been supported by a vast number of recent neuroimaging as well as lesion studies, showing that CM is associated with large [17], midline [18] tumours residing in proximity of the fourth ventricle and predicted by damage of the superior cerebellar peduncles, the dentate nucleus or the CDTC pathway itself [19]. Although tractography can improve preoperative surgical planning or predict postoperative deficit [19], its intraoperative reliability is limited by tissue displacement and brain-shift phenomena [20]. Currently, no intraoperative method exists that can provide a mean to CDTC pathway preservation.

We recently reviewed all intraoperative studies aimed at mapping the cerebellum, and, while CM has been extensively explored using neuroimaging techniques, neurophysiological reports are lacking [21]. This may be surprising, considering that intraoperative neurophysiological monitoring (IONM) is particularly well developed for other posterior fossa surgeries. However, as the cerebello-dento-thalamo-cortical pathway is polysynaptic, it differs from direct motor connections which are less influenced by anaesthesia [22]. One way to assess the CDTC circuit has been provided by non-invasive brain stimulation. Cerebellar stimulation by means of transcranial electrical stimulation (TES) or by transcranial magnetic stimulation (TMS) modulates the amplitude of motor-evoked potentials (MEPs) following TMS of the motor cortex [23]. Such modulation is commonly inhibitory—though it can take the form of excitation with appropriate TMS parameters—and is commonly referred to as cerebellar inhibition (CI) [23]. The latency at which such inhibition occurs is of 5–8 ms, with cerebellar stimulation as conditioning stimulus (cStim) occurring before the test stimulus (tStim) over the motor cortex. CI is commonly thought to be a result of activity along the CDTC pathway [23, 24]. Noticeably, this phenomenon is absent in cases of damage to the CDTC pathway, such as a lesion to the cerebellar hemispheres, the dentate nucleus, the superior cerebellar peduncle and the motor thalamus [25]. We speculate that the adoption of a similar paradigm intraoperatively may offer a method to identify, and possibly prevent, cerebello-dento-thalamo-cortical pathway disconnection and therefore cerebellar mutism [21]. With this aim, we explored the feasibility of using an intraoperative paired cerebello-cortical stimulation in ten patients undergoing posterior fossa surgery.

## Methods

### Patients’ cohort

The study proposal is in accordance with ethical standards of the Declaration of Helsinki. All stimulations and recordings were performed in the context of clinical intraoperative neurophysiological monitoring (IONM). Patients scheduled for posterior fossa surgery were screened for enrolment and signed a written consent to adhere. The inclusion criteria were (1) posterior fossa disease with indication to intraoperative neurophysiological monitoring. Exclusion criteria were (1) voluntary decision of the patient or his/her family not to be included in the cohort. Ten patients (age 6–73; 5M-5F; 10 right-handed, 2 children) were included in this study. Patient’s characteristics are presented in Table 1.

**Table 1:** Patient demographics

Patient	Age	Sex	Symptoms	Diagnosis	Surgical approach
#1	73	F	Incidental	Tentorial meningioma (WHO I)	RS
#2	47	F	Vertigo	Tentorial meningioma (WHO I)	MS
#3	8	M	Hydrocephalus	Medulloblastoma (WHO IV)	MS
#4	23	M	Cephalalgia	Chiari malformation I	MS
#5	37	M	Diplopia	Glio-neuronal neoplasia of the lamina quadrigemina (WHO III)	MS
#6	37	M	Vertigo	Hemangioblastoma in VHL	RS
#7	50	M	Vertigo	Hemangioblastoma in VHL	MS
#8	61	F	Hear loss	Vestibular schwannoma (WHO I)	RS
#9	18	F	Cephalalgia	Hemangioblastoma in VHL	MS
#10	9	M	Hydrocephalus, diplopia	Pilocytic astrocytoma (WHO I)	MS

*MS* median suboccipital approach, *RS* retrosigmoid approach, *VHL* Von Hippel-Lindau syndrome

### Stereotaxic neuronavigation and electrode placement

MRI scans of each patient’s brain were acquired before surgery on a 1.5T or 3T scanner with an eight-channel head coil (Signa *3T*, *General Electric* Healthcare, Milwaukee, USA). T1-weighted 3D MPRAGE images were acquired using the following parameters (echo train length: 1, TE: 2.67 ms, TR: 2.000, matrix size: 256 × 246, slice thickness: 1 mm). T2-weighted, FLAIR images were also acquired (TR 6000 ms, TE 150 mss, TI 2000 ms). The reconstruction of the individual cortical surface was performed using Brainsuite (Brainsuite [26], UCLA Brain Mapping Center, San Francisco, USA). For a clearer intraoperative visualization of sulcal anatomy, a skull stripped T1 using a non-uniformity correction or FLAIR images was added to the 3D visualization of the Neuronavigation system (Stealth Station 7, Medtronic, Minneapolis, USA). Correspondence of 3D reconstruction and individual patient’s sulcal anatomy was then performed using the Neuronavigation pointer. Brain anatomy was systematically analysed prior to surgery so that the main sulcal pattern of the principal sulcus as well as the vermis could be identified during surgery. Placement of the conditioning electrode strip was planned *a priori* but was systematically reprogrammed when in presence of contingent surgical conditions preventing the placement of the strip in the desired position, such as presence of large vessels or space requirements by the ongoing surgical procedures.

### Anaesthesia and conventional IONM

The anaesthesia protocol applied was Total IntraVenous Anaesthesia (TIVA). More precisely, a continuous infusion of Propofol (100–150 μg/kg/min) and Fentanyl (1 μg/kg/min) was used, avoiding bolus. Halogenated anaesthetic agents were never used. Since all patients were candidates for IONM of the corticospinal tract, standard neurophysiological monitoring and mapping were performed. This involved simultaneous acquisition of continuous electroencephalography (EEG) and recording of free-running electromyographic (EMG) activity (ISIS-IOM, Inomed Medizintechnik GmbH, Emmendingen, Germany). Muscle MEPs were elicited by transcranial electrical stimulation (TES) via corkscrew-like electrodes (Ambu Neuroline Corkscrew, Ambu, Copenhagen, Denmark) from the scalp. Short trains of 5 square-wave stimuli of 0.5 ms duration and interstimulus interval (ISI) of 2 ms were applied at a repetition rate up to 2 Hz through electrodes placed at C1 and C2 scalp sites, according to the 10/20 EEG system [27].

### Motor stimulation as test stimulus

**Fig. 1:**
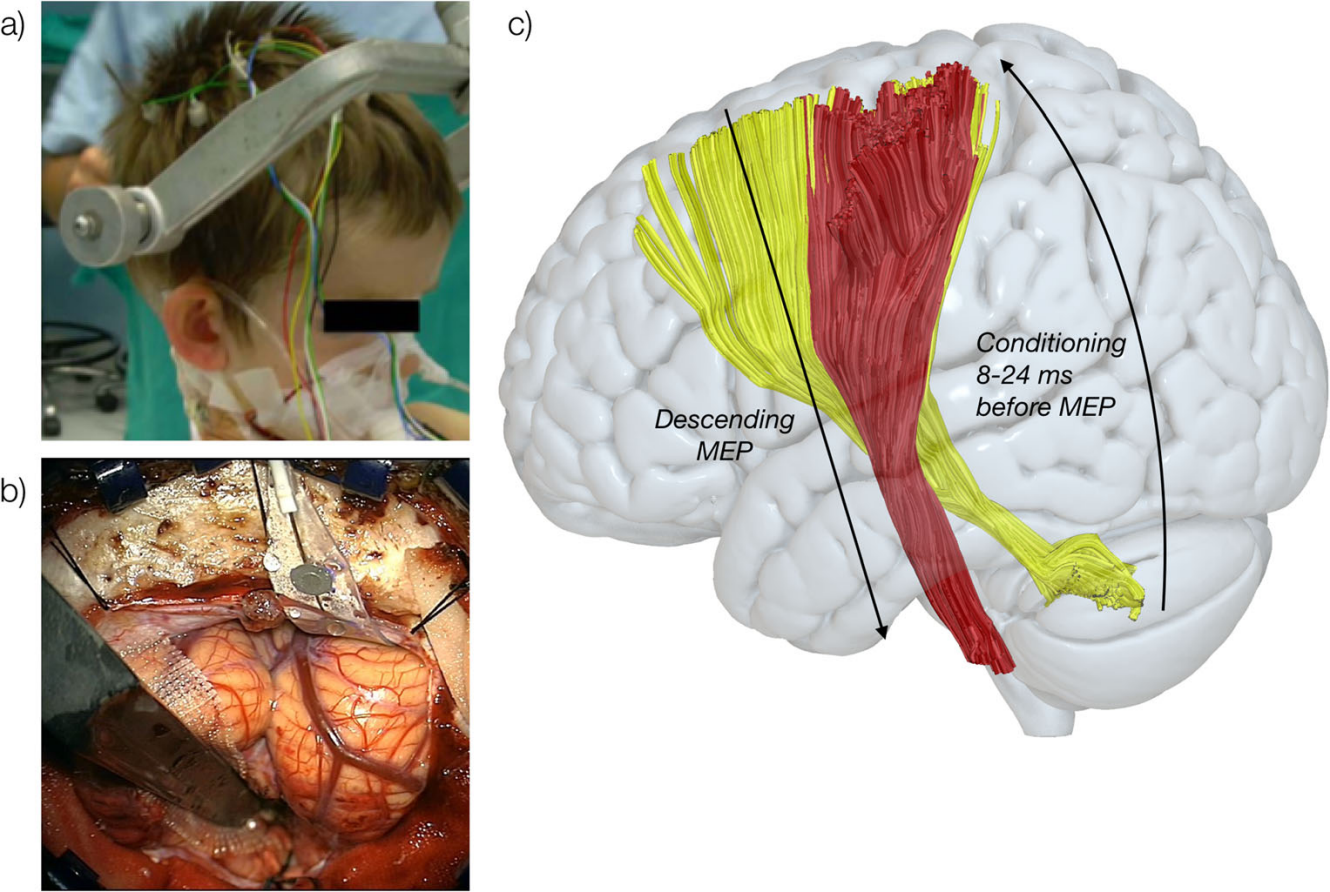
Schematic representation of the stimulation protocol. **a**) M1 stimulation (test stimulus) is performed using transcranial electric stimulation using a C1-C2 dipole on the skull, which generates motor-evoked potentials by activating the corticospinal tract. **b**) Direct cerebellar stimulation is applied as conditioning stimulus prior to M1 stimulation, causing MEP modulation. **c**) Representation of stimulation protocol: direct cerebellar precedes transcranial electrical stimulation with an inter-stimulus interval (ISI) between 8 and 24 ms. Conditioning stimuli generating MEP facilitation/inhibition compared with test stimuli alone are considered evidence for functional connectivity between the two stimulated regions. Red: corticospinal tract; yellow: cerebello-dento-thalamo-cortical pathway

TES was applied to precentral gyrus (test stimuli) via a C1-C2 dipole on the skull using corkscrew (Ambu Neuroline Corkscrew, Ambu, Copenhagen, Denmark) (Fig. 1 shows a schematic of the dual stimulation protocol and an example of surgical scenario). Stimulation parameters were personalised in each patient and set to obtain a MEP from the thenar muscle of around 500 μV peak-peak amplitude (see Table 2). As a result, stimuli were delivered with trains of 2–5 stimuli in different patients, at an intrinsic frequency of 500 Hz at an intensity of 75–200 mA (Table 2).

**Table 2:** Stimulation parameters

Patient	No pulses	TES (mA)	DCS (mA)	MEP from direct cerebellar DCS	Pre-operative MRC	Post-operative MRC	Follow-up MRC
#1	To3	140	20	no	5	5	5
#2	To4	180	15	no	5	5	5
#3	To5	180	25	no	5	2	5
#4	To5	160	20	no	5	5	5
#5	To5	200	20	no	5	5	5
#6	To5	200	20	no	5	5	5
#7	To2	180	20	no	5	5	5
#8	To3	90	20	no	5	5	5
#9	To4	75	20	no	5	5	5
*#10*	To5	190	20	no	5	3	5

*TES* transcranial electrical stimulation, *DCCS* direct cerebellar cortical stimulation, *MRC* Medical Research Council

### Cerebellar stimulation

The cerebellar cortex was stimulated with a short train of stimuli at 500 Hz ranging from 2 (To2) to 5 (To5), with current intensity of 15–25 mA (Table 2). Stimuli were delivered through an electrode strip (contact diameter: 5 mm, interelectrode distance of 10 mm, contact strips: 0.7 mm thin, 10 mm width, EB Neuro S.p.A., Firenze, Italy) placed directly on the cerebellar cortex where the cortex was exposed and gently slipped under the dura outside the craniotomic window. Bipolar stimulation was delivered using pairs of adjacent electrodes. The number of effective contacts varied between patients according to surgical needs.

### Paired stimulation protocol

Paired stimulation was compared with M1-only stimulation. Briefly, we acquired 20 stimulation from transcranial electric stimulation of M1, which were considered as baseline stimuli. Then, other 20 stimuli were acquired directly stimulating the cerebellum alone, in order to exclude any direct MEP result arising from the cerebellum. To conclude, we performed 20 paired stimulations where the cerebellum was stimulated before M1. M1 stimulation occurred therefore after cerebellar stimulation at fixed interval between 8 and 24 ms. The timing of dual stimuli was managed entirely by the commercially available ISIS-IOM system (Inomed Medizintechnik GmbH, Emmendingen, Germany) by means of the “facilitation” function, which allows independent electrical stimulation through two separate output channels.

### Data analysis

Data analysis is explained in detail in the Supplementary Material. Briefly, paired stimulation was compared with M1 only stimulation (baseline stimulation) in the single patient. Pre-processing required the data to be exported in digital format and analysed with MATLAB software. The EMG traces were band-pass filtered (10–3000 Hz) and positive peak-to-peak amplitude was extracted from each MEP. As data were not normally distributed in blocks of stimuli, a non-parametric Mann-Whitney’s *U* test was performed. Paired stimulation that leads to a significant increase in MEP compared with baseline was considered excitatory, while those that lead to a significant decrease in MEP were considered inhibitory. The significance threshold for the *p* value was set to 0.005 rather than performing actual multiple comparison correction, as the number of conditions (strip electrodes and ISIs) was not the same in all patients, and accordingly, the number of multiple comparisons varied among participants. Individual cerebellar anatomy and stimulation sites acquired during neuronavigation were normalised to the MNI space using SUIT[28] (http://www.diedrichsenlab.org). Since not all stimulation sites could be acquired with neuronavigation because it is not exposed in the craniotomy, the location of each subdural electrode was reconstructed using 3D Slicer (www.slicer.org) using the strip electrode trajectory on individual cerebellar surface considering a fixed interelectrode distance of 1 cm. MNI coordinates of stimulation sites per individual patient can be found in Table 3.

**Table 3:** MNI location of all recording contacts

Patient	Recording electrodes	MNI coordinates		
#1	EL	*x*	*y*	*z*
	1	51	− 50	− 51
	2	51	− 61	− 51
	3	47	− 71	− 50
	4	40	− 80	− 46
#2	EL	*x*	*y*	*z*
	1	25	− 88	− 24
	2	24	− 91	− 31
	3	19	− 92	− 39
	4	14	− 89	− 45
#3	EL	*x*	*y*	*z*
	1	26	− 57	− 15
	2	26	− 67	− 16
	3	24	− 77	− 18
	4	23	− 85	− 21
#4	EL	*x*	*y*	*z*
	1	18	− 65	− 11
	2	21	− 72	− 15
	3	25	− 80	− 19
	4	30	− 88	− 24
#5a	EL	*x*	*y*	*z*
	1	5	− 76	− 49
	2	12	− 73	− 57
	3	20	− 69	− 61
	4	30	− 63	− 64
	5	36	− 54	− 63
	6	38	− 44	− 57
#5b	EL	*x*	*y*	*z*
	1	17	− 92	− 31
	2	27	− 91	− 32
	3	37	− 87	− 32
	4	45	− 81	− 33
#6a	EL	*x*	*y*	*z*
	1	26	− 47	− 15
	2	32	− 57	− 18
	3	39	− 66	− 20
#6b	EL	x	y	z
	1	16	− 89	− 45
	2	12	− 91	− 37
#7	EL	*x*	*y*	*z*
	1	16	− 49	− 9
	2	20	− 59	− 10
	3	23	− 67	− 15
#8	EL	*x*	*y*	*z*
	1	18	− 42	− 11
	2	21	− 52	− 12
	3	24	− 62	− 14
	4	28	− 71	− 18
	5	33	− 80	− 21
#9	EL	*x*	*y*	*z*
	1	9	− 78	− 54
	2	17	− 77	− 58
	3	25	− 72	− 61
	4	32	− 65	− 63
	5	37	− 56	− 62
#10	EL	*x*	*y*	*z*
	1	18	− 42	− 11
	2	21	− 52	− 12
	3	24	− 62	− 14
	4	28	− 71	− 18
	5	33	− 80	− 21
	6	18	− 42	− 11

NB. Each patient’s second strip positioning in a is signalled as a “b”

## Results

**Fig. 2:**
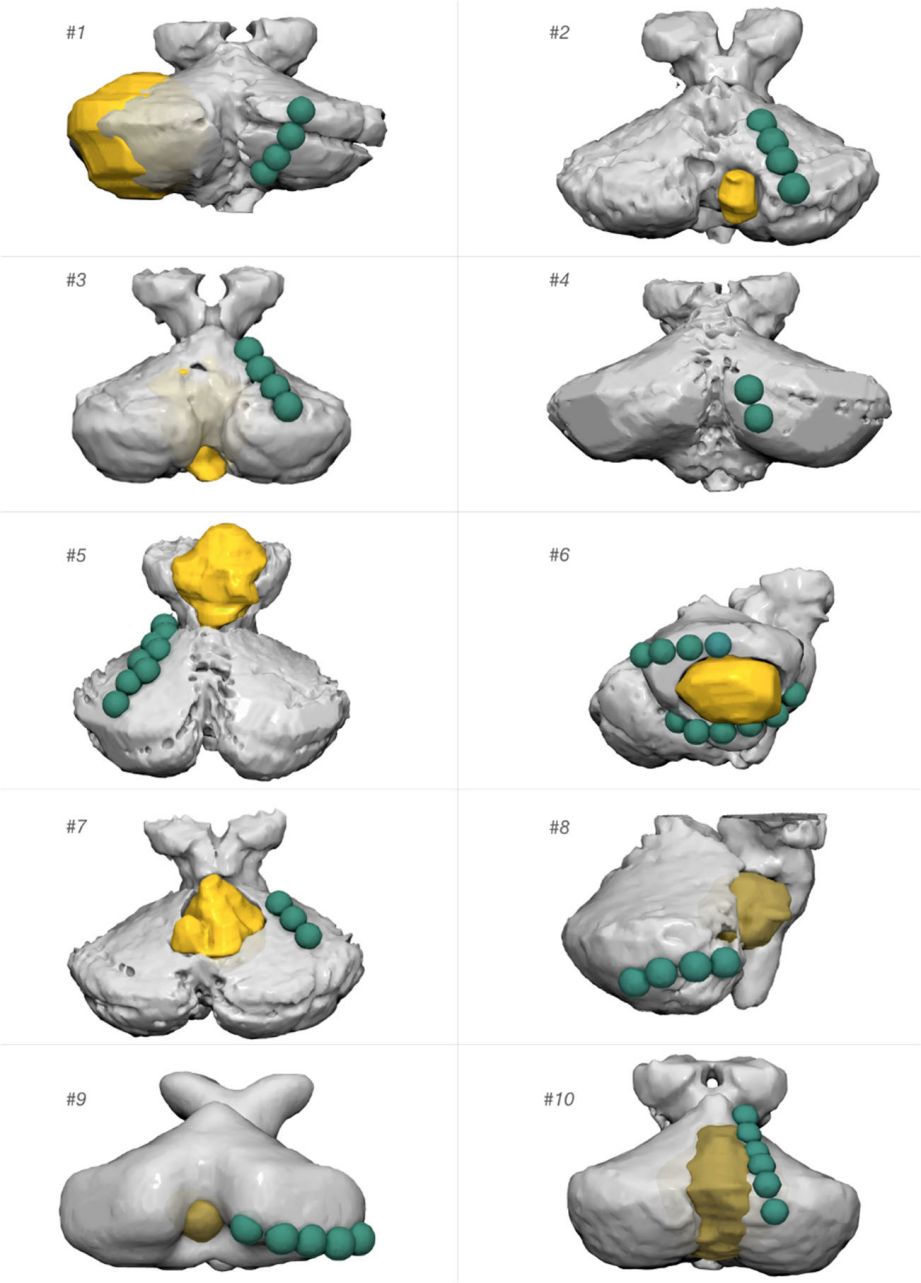
Rendering of the individual cerebelli. The projection of the lesion on the surface is displayed in yellow. The individual stimulation sites are shown in green (note that conditioning stimuli have been delivered in bipolar modality)

**Fig. 3:**
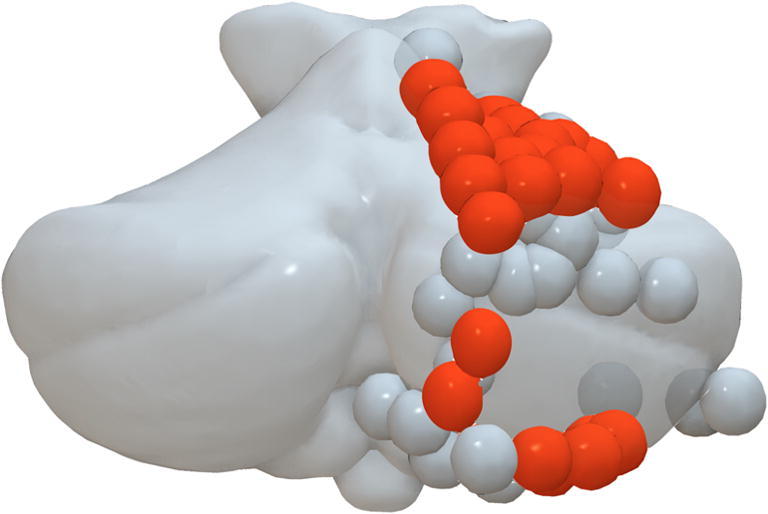
Normalised data from all patients. All sites are displayed on the right hemisphere. Effective sites are shown in orange, occupying the intermediate portion of lobule IV, V, VI and VIIB. Ineffective sites are shown in grey

In all participants, it was possible to stimulate at least one conditioning dipole. Figure 2 shows each patient’s anatomy together with lesion location and electrode placement while Fig. 3 shows each electrode site normalised to the MNI space and visualised on the right side. Intensity of stimulation and conditioning as well as clinical outcome is shown in Table 2.

### Direct cerebellar stimulation alone

No MEP was evoked with direct cerebellar stimulation in any of the patients.

### Paired cortico-transcortical stimulation

We observed significant modulation of MEPs in 8/10 patients. Five patients showed MEP inhibition, 1 patient MEP facilitation and 2 patients showed both conditions at different ISIs. The individual results are reported in the Supplementary Table 1. Coordinates for strip electrodes normalised to MNI can be found in Table 3.

### Anatomical localization of conditioning effects

Group analysis was not quantitatively performed, and therefore, any observation on the anatomical location of effective cerebellar stimulations is descriptive. Overall, two clusters of response were evidenced: a larger cluster occupying the intermediate portion of lobule IV, V and VI in the anterior cerebellum, and a smaller cluster in the posterior cerebellum occupying the intermediate pre-pyramidal fissure and lobule VIIB. When comparing inhibitory and facilitatory effects in the anterior cerebellum, these seemed to overlap in the anterior cerebellum, though inhibitory responses seemed to be more widespread in both clusters. In the posterior cerebellum, no sites for MEP facilitation were evidenced.

### Chronometry of conditioning effects

**Fig. 4:**
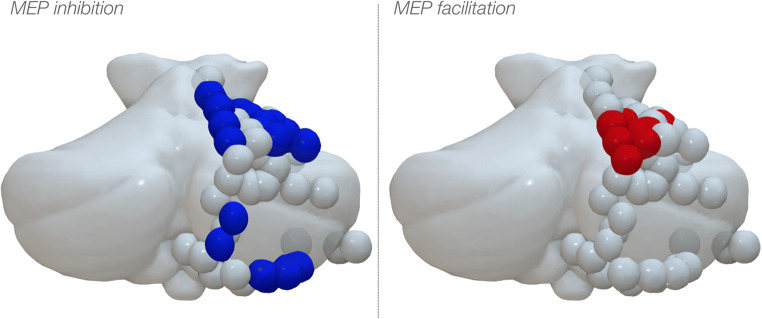
Standardised cerebellar anatomy showing the location of each participant’s conditioning electrodes in the cerebellar cortex. All stimulation sites have been displayed on the right hemisphere as spheres. The left panel shows the cerebellar location of dipoles with inhibitory effect over M1, occurring when conditioning over the intermediate lobule IV-V-VI in the anterior cerebellum or the lobule VIIB in the posterior cerebellum. The right panel indicates the locations where conditioning caused MEP facilitation, covering the intermediate lobule IV-V-VI in the anterior cerebellum. Blue-filled spheres indicate spots with significant inhibitory conditioning effects. Red-filled symbols indicate spots with significant excitatory conditioning effects. Grey-filled symbols indicate spots with no significant effect

Conditions of significant inhibition or excitation were not univocally associated with a single ISI, though a prevalence for the 8 ms ISI was observed. Inhibitory responses were found overall in 14/152 positions (9%). These were distributed between different ISIs as follows: in 3/13 (23%) positions at the 8 ms ISIs, in 2/36 (6%) positions at the 12 ms ISI and at 5/36 positions (14%) at the 16 ms ISI, in 3/33 positions (9%) at the 20 ms ISI and in 1/26 positions (4 %) at the 24 ms ISI. Excitatory responses were much less frequent, appearing only in 4/152 positions, none of which at 8 ms and one for each of the other 4 ISIs. The group analysis yielded significant results (H (4, *N* = 144) = 11.57 *p* = 0.021). The results are indicated in Fig. 4. Post hoc exploration of the significant results was carried out with Mann-Whitney’s *U* tests, comparing the data from each ISI reciprocally. The results indicated that the main effect was entirely due to the 8 ms ISI having lower values than all other ISIs. In particular, the 8 ms ISI was significantly different from the 12 ms ISI (*Z* = − 2.93; *p* = 0.003), from the 16 ms ISI (*Z* = − 3.04; *p* = 0.002), from the 20 ms ISI (*Z* = − 2.66; *p* = 0.008) and from the 24 ms ISI (*Z* = − 2.81; *p* = 0.004). All other reciprocal comparisons between all other ISIs were non-significant. Finally, as a second post hoc comparison, we sought to understand whether the data from each ISI was significantly different from a distribution centred on a mean value of *x* = 0. The significance of this test was to assess whether stimulation at each ISI yielded significant inhibition, no effect or facilitation. The results showed that the *z*-values in the 8 ms ISI were highly significantly smaller than *x* = 0 (*t*(12) = − 5.57; *p* = 0.0001), corresponding to MEP inhibition, while at all other ISIs, no effect was observed (min *p* value = 0.17).

**Fig. 5:**
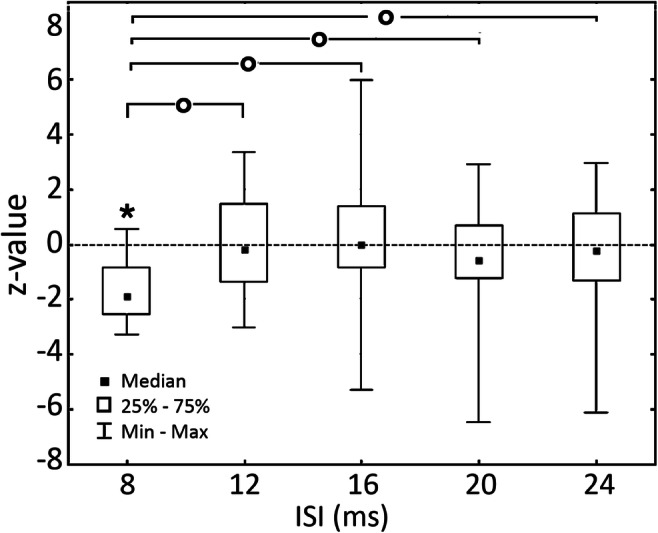
*z* values from the whole population of patients, grouped by ISI. Circles indicate significant differences between ISIs. The asterisk indicates the significant difference between the data in each ISI and the null hypothesis of a distribution centred on *x* = 0

**Fig. 6:**
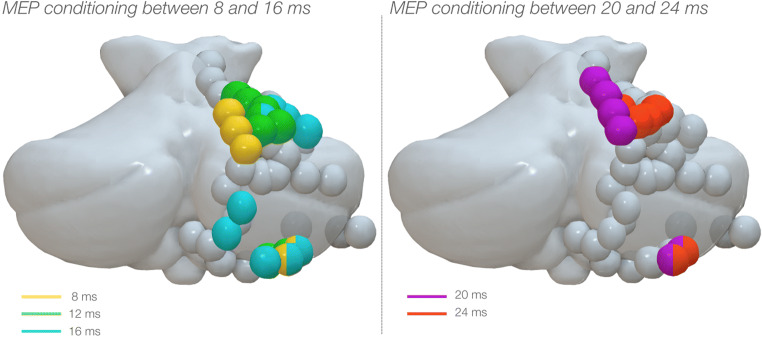
Standardised cerebellar anatomy showing the location of each participant’s conditioning electrodes in the cerebellar cortex. All stimulation sites have been displayed on the right hemisphere as spheres. The left panel shows with cerebellar location of dipoles with conditioning effect over M1 at ISI between 8 and 16 ms. A medio-lateral distribution was displayed in the anterior cerebellum, with shorter ISI lying medially and longer ISI. Mixed distribution was shown in the inferior cerebellum. ISI at 8 ms: yellow dots; ISI at 12 ms: green dots; ISI at 16 ms: light blue dots. The right panel indicates as purple dots the locations where conditioning responses were evoked at 20 ms and orange dots at 24 ms

The anatomical location of the conditioning spots that exerted a modulation on the MEPs is illustrated in Fig. 5. Qualitative inspection seemed to indicate defined clusters for the different ISIs. In particular, a medio-lateral distribution of conditioning responses was shown in the anterior cerebellum: 8 ms responses occurred medially, 12 ms were central and 16 ms responses lateral. Similarly, 20 ms lied medially to those at 24 ms. Responses in the posterior cerebellum showed mixed distribution (Fig. 6).

## Discussion

We explored the feasibility of intraoperative dual cerebello-motor stimulation with the aim of developing an intraoperative method to monitor, and therefore preserve, the cerebello-dento-thalamo-cortical pathway, a principal network involved in cerebellar mutism. To do this, we reproduced the extraoperative conditioning-test paradigm developed by Ugawa and colleagues [23] intraoperatively.

In agreement with previous extraoperative literature [23], our data showed a clear inhibition when cerebellar stimulation preceded M1 stimulation of 8 ms. However, conditioned responses with an ISI longer than 12 ms were also identified, showing mixed inhibitory and facilitatory effects. Responses showed anatomical selectivity for the intermediate hemispheric lobule IV-V-VI in the anterior cerebellum and for the mid-lateral portion of lobule VIIB in the posterior cerebellum. In summary, we show that cerebello-cortical stimulation is feasible intraoperatively under general anaesthesia, with inhibitory responses at 8 ms likely representing the CDCT pathway. We speculate that using this cerebello-cortical paradigm during surgery could be used to predict, and potentially prevent, CDCT disconnection in children operated in the posterior fossa. This may help reduce postoperative cerebellar mutism and improve children’s quality of life.

### Anatomical specificity for cerebello-cortical stimulation

Dual coil TMS is a well-established technique for proving cerebellar function, which is characterised by MEP inhibition when cerebellar stimulation proceeds M1 stimulation about 5–8 ms [29]. To reproduce non-invasive stimulation results, we combined direct cerebellar stimulation with transcranical electrical stimulation using a 10–20 EEG setting [30], modifying a paradigm developed for supratentorial brain surgery [31]. MEP conditioning showed a very clear location specificity: MEP modification occurred when conditioning stimulations were performed in the intermediate lobule IV-V-VI in the anterior cerebellum and in the intermediate lobule VIIB in the posterior cerebellum. Strikingly, the anatomical location of our cerebellar maps corresponded with cerebellar activation for hand movement using fMRI found by Grodd and colleagues [32] in both the anterior and posterior cerebellum. Cerebellar stimulation caused MEP inhibition, reproducing established phenomena of the extraoperative setting [23, 24, 29]. However, areas of MEP facilitation were also shown. This is consistent with data of non-invasive protocols in humans, showing excitatory cerebellar effects on M1 depending on stimulation parameters [23] and with animal studies showing that the cerebellum modulation would involve both cerebellar inhibition and excitation [33, 34], since excitatory pathways exist that project to the cortex through the ventral thalamus [33]. Patterns of excitation and inhibition seemed to overlap in the anterior cerebellum, though inhibitory responses were more numerous and thus seemed more widespread. Facilitation was not evinced in the posterior cerebellum; however, it was less tested in our cohort compared with the anterior cerebellum. One issue that deserves discussion is the focality of cerebellar stimulation. We delivered bipolar stimuli through a dipole of 5 mm electrodes, 10 mm apart, at variable intensities, in the 10–25 mA range. We know from empirical and simulation data that inform us that stimuli in the cerebral cortex using these parameters produce electrical fields that are focal [35, 36]. We can safely assume that the electrical field to bipolar stimulation is similar to that obtained by cerebral cortical stimulation; therefore, efficient neural stimulation did not occur in distant structures such as the cerebellar nuclei or the brainstem. However, it is uncertain how the electrical field translates into an electrical current and impacts the function of nervous tissue. Indeed, the cerebellar cortex shows striking anatomical differences from the cerebral cortex, including thinner cortical layers, deeper folding, dense stacking of adjacent folds and a much smaller radius of curvature of the surface. All these features add complexity to the problem of focality of stimulation. Given the complex folding pattern of the cerebellar cortex, it is likely that stimulation involves both cortical axons and efferent (Purkinje) axons in the white matter and that the cortex has been stimulated in an irregular and patchy pattern, with sparing of the deep part of the cerebellar sulci. A specific prediction on the local topography of stimulation can be made only by means of electrical-field modelling in anatomically realistic models of the cerebellar cortex, which are currently not available in the literature.

### Chronometry of cerebello-cortical stimulation

Responses showed defined clusters which were ISI-related. In the anterior cerebellum, modulation showed a medio-lateral gradient, with 8 ms responses lying most medial and others lying progressively more lateral until 16 ms. Moreover, responses at 24 ms lied lateral to those at 20 ms. In the posterior cerebellum, conditioning responses had a more mixed distribution. Responses at 8 ms were always inhibitory in our cohort. This is consistent with the TMS literature, where cerebellar stimulation classically causes inhibition [29]. However, for all other intervals, inhibition nor facilitation was ISI-specific. This differs from Iwata and colleagues [37], where facilitation was associated with 2 ms conditioning only. As modulation from the cerebellum to the cerebrum should occur as fast as 5–8 ms [23], potentials occurring at 8 ms should represent the CDTC pathway. Responses at longer intervals (12–24 ms) may be consistent with other mechanism, possibly comprising activation of cerebello-spinal pathways [23]; however, further research is needed to elucidate involved connectivity.

### Cerebello-cortical stimulation and surgical relevance

There is currently no method to monitor the CDTC pathway, considering that awake surgery is not indicated in pediatric patients [38], with 50-Hz stimulation technique being scarcely effective on children [39]. For the first time to our knowledge, we described this technique with the specific aim of providing a way to monitor the CDTC pathway and possible lower the incidence of CM. The present results are preliminary and exploratory. To capitalise on our results for the purpose of refining an intraoperative monitoring technique, it is probably better to focus on conditioning (cerebellar) stimuli applied to the intermediate lobule IV-V-VI in the anterior cerebellum or intermediate lobule VIIB, with ISIs around 8 ms or less (we did not manage to investigate ISIs < 8 ms in a systematic way), expecting purely inhibitory effects on the corticospinal motor pathway. Further studies using this method as a monitoring technique are needed to validate its reproducibility. In addition, the value of this potentially innovative neuromonitoring technique remains to be validated in the clinical setting, as this was not the goal of our feasibility study. We aim now to prospectively correlate neuromonitoring data (presence or absence of M1 output inhibition) with the clinical outcome in pediatric posterior fossa tumour surgery.

Finally, there is evidence that posterior fossa tumour resection can have different effects on children with pre-existing language impairment, which may represent a subclinical state of CMS in some children with posterior fossa tumour [40]. In this perspective, pre-operative TMS, following the protocol suggested by Ugawa and colleagues [23], may offer the opportunity to confirm the presence of a pre-surgical impairment of the CDTC pathway in these patients.

## Limitations

There are several limitations to this study. First, the study collects both pediatric and adult patients: however, while CM is classically shown in children, CDTC is represented in both patients. Second, we could not monitor spinal excitability (e.g. with the H-reflex or F-waves) to distinguish whether conditioning occurring with a longer ISI represented cerebello-cortical or cerebello-spinal efferents. This issue should be addressed in further studies.

## Conclusion

We showed for the first time that cerebello-cortical stimulation is a feasible technique to evidence cerebellar connectivity. While our results are compatible with previous literature on response chronometry, they also show a pronounced anatomical specificity for cerebellar modulation occurring in the mid-portion of hemispheric lobule IV-V-VI in the anterior cerebellum or in the second third of lobule VIIB, with both inhibitory but also excitatory effects evoked. These results may offer a new intraoperative technique for children suffering from posterior fossa tumour, with the aim to further contribute to prevent neurological deficits and hence to preserve quality of life.
